# The use and usefulness of point-of-care tests in patients with pharyngotonsillitis – an observational study in primary health care

**DOI:** 10.1186/s12875-023-02245-9

**Published:** 2024-01-06

**Authors:** Jon Pallon, Martin Sundqvist, Katarina Hedin

**Affiliations:** 1 Department of Clinical Sciences in Malmö, Family Medicine, Lund University, Malmö, Sweden; 2 Department of Research and Development, Region Kronoberg, Växjö, Sweden; 3 Department of Laboratory Medicine, Clinical Microbiology, Faculty of Medicine and Health, Örebro University, Örebro, Sweden; 4 Department of Health, Medicine and Caring Sciences, Futurum, Region Jönköping County, Linköping University, Linköping, Sweden

**Keywords:** Pharyngotonsillitis, Rapid antigen detection test, C-reactive protein, Aetiology, Primary health care, Antibiotic prescribing

## Abstract

**Background:**

Rapid antigen detection tests (RADT) for Group A streptococci (GAS) and point-of-care tests for C-reactive protein (CRP) are commonly used in patients with pharyngotonsillitis in Sweden and Denmark although CRP testing is not supported by guidelines. We aimed to describe (1) the proportion of patients tested with RADT and/or CRP, (2) the relation between test results and antibiotic prescribing, and (3) the association between CRP level and microbial aetiology.

**Methods:**

We used a post-hoc-analysis of data collected in primary health care in a prospective aetiological study of 220 patients 15–45 years old diagnosed with pharyngotonsillitis. The outcomes of RADTs and CRP tests were related to antibiotic prescribing and microbial aetiology.

**Results:**

A RADT was used in 94% of the patients. A CRP test was used in 50% of the patients but more commonly in those with a negative RADT (59%) than in those with a positive RADT (38%) (*p* = 0.005). Most (74%) CRP tests were used in patients with a negative RADT. Antibiotic prescribing differed greatly between patients with a positive RADT (96%) and patients with a negative RADT (17%) (*p* < 0.001). In patients with a negative RADT, there was a positive association between CRP value and antibiotic prescribing (OR 1.05; 95% CI 1.02–1.07; *p* < 0.001). Patients with CRP values ≤ 30 mg/l were seldomly prescribed antibiotics. Patients with GAS in culture had the highest median CRP (46 mg/l), which was higher than in patients without GAS (8 mg/l; *p* < 0.001). However, the positive predictive value for GAS never exceeded 0.60 (95% CI 0.31–0.83) at the investigated CRP levels.

**Conclusions:**

The widespread use of tests is a major deviation from national guidelines. Most CRP tests were used in patients with a negative RADT, suggesting a belief in the added value of a CRP test, and the CRP result seemed to influence antibiotic prescribing. However, as an aetiological test, CRP is not useful for predicting GAS.

## Background

Acute sore throat – pharyngotonsillitis – is one of the most common infections in primary health care (PHC) [1, 2]. Respiratory viruses are thought to cause the majority of pharyngotonsillitis cases [3, 4] but *Streptococcus pyogenes* (group A streptococci; GAS) is the most important pathogen and the only one that warrants antibiotic prescribing according to most guidelines [5]. Other bacteria that have been suggested as pathogens include *Streptococcus dysgalactiae* ssp. *equisimilis* (also known as group C and G streptococci) [6, 7] and *Fusobacterium necrophorum* [8, 9]. GAS is detected in 14% of adults [10] and 37% of children [11] with a sore throat, and most sore throat guidelines recommend the use of a clinical scoring system, e.g., the Centor score, to help identify these patients [5, 12]. However, as the predictive values of these systems are only moderate [13, 14] many guidelines recommend subsequent point-of-care (POC) testing with a rapid antigen detection test (RADT) for GAS for patients with high scores [5]. In Sweden, the national guideline recommends a RADT in patients with a Centor score of 3–4 [15]. RADTs are both highly sensitive and specific [16, 17] but cannot separate infection from an asymptomatic carriage. The use of RADTs might lower the antibiotic prescribing by 25% if negative results are used to refrain from prescribing [18], and in many European countries the prescribing for respiratory tract infections is negatively correlated with the use of POC tests [19]. On the other hand, there is a concern that RADTs could be overused and that treatment decisions will be based on test results alone rather than on the severity of symptoms [14, 20]. In addition, testing could have a medicalising effect on patients and increase their demand for RADTs in future episodes of sore throat [14].

C-reactive protein (CRP), a marker of inflammation, has a long history of use in hospitals as a means of differentiating bacterial from viral infections [21]. However, its discriminatory ability is limited as viral infections can also give rise to high CRP levels [21–23]. In PHC, there is some evidence that POC testing with CRP might heighten the diagnostic accuracy of lower respiratory tract infections and lower the antibiotic prescribing [24–26]. Only a few studies have looked at CRP in pharyngotonsillitis [27–34]. Although some of these point to higher levels in patients with GAS [27–31, 35], the positive predictive value (PPV) of CRP is very low [35], especially in low-prevalence settings such as PHC [10]. Moreover, no study has investigated whether CRP can identify patients who would benefit from antibiotic treatment. Globally, no sore throat guideline recommends CRP testing [5]. Some authors, however, claim that CRP could lower prescribing by as much as half in low-resource settings that lack RADTs, using certain threshold values for treatment [27–29, 36].

The Nordic countries have a long history of POC testing with RADTs and CRP, and these tests are now routinely used in PHC. However, the proportion of patients who are tested is much larger than expected from guidelines, and this pattern is stable over time [24, 32, 37, 38]. A recent study found that 48% of Danish general practitioners are still using CRP and/or leukocytes in patients with pharyngotonsillitis, despite 96% of them also using RADT [37]. In line with this, another study found that 56% of Danish patients with a sore throat are tested with CRP [24].

This study used a post-hoc analysis of pre-COVID-19 data collected for a prospective aetiological study on pharyngotonsillitis in Swedish PHC [9], to investigate (1) the proportion of patients tested with RADT and/or CRP, (2) the association between POC test results and antibiotic prescribing, and (3) the association between CRP level and microbial aetiology.

## Methods

### Study setting

This cross-sectional study of young adults with pharyngotonsillitis in PHC is a post-hoc analysis of data originally collected for an aetiological study in Region Kronoberg, Sweden, during two winter half-years (2011–12) [9]. Region Kronoberg, located in southern Sweden, has a population of 190,000, and is served by two hospitals and 34 PHC centres. Five of these PHC centres took part in the study and were chosen by convenience.

### Population

The participating PHC centres used telephone triage nurses to make a first assessment of the medical needs of the patients. Patients aged 15–45 years with an acute sore throat as a major complaint and who were judged to require a doctor’s visit were asked to participate. The Swedish guideline states that patients with compelling signs of viral infection should stay at home and use self-care and that only patients with 3–4 Centor criteria and a possible benefit from antibiotics should be tested with an RADT [15]. If the doctor confirmed infectious pharyngotonsillitis, the patient was recruited after giving informed consent. We aimed for a consecutive sample during the two inclusion periods but had to settle with a convenience sample. In total, 220 patients were included.

### Clinical data

The participating doctors were instructed to manage the patients as they would normally do, with the addition of registering clinical data from the visit about background characteristics, medical history, signs, symptoms, diagnosis, tests and treatments.

### Microbiological sampling and procedures

After seeing the doctor, all participating patients were sampled from the throat, nasopharynx, and blood by trained laboratory staff and screened for 20 pathogens [9, 39]. Routine culture was used to detect β-haemolytic streptococci (Lancefield group A, C, and G), anaerobic culture was used to detect *F. necrophorum*, serology was used to detect Epstein–Barr virus, single PCR was used to detect Influenza A and B viruses and *Mycoplasma pneumoniae*, and multiplex real-time PCR was used to detect the intracellular bacteria *M. pneumoniae* and *Chlamydophila pneumoniae* and the viruses Adenovirus, Bocavirus, Coronavirus NL63, Coronavirus OC43, Coronavirus HKU1, Coronavirus 229E, Enterovirus, Influenza A virus, Influenza B virus, Metapneumovirus, Parainfluenzavirus, Rhinovirus, and Respiratory syncytial virus [39].

### Point-of-care-tests

RADTs and CRP tests are widely used in Swedish PHC and are readily available at most PHC centres. In Region Kronoberg, the RADT kit used during the study period was Quick-Vue Dipstick Strep A (Quidel Corporation, San Diego, CA, USA), a lateral-flow immunoassay using antibody labelled particles. The CRP test used during the study period was Afinion™ CRP assay (Abbott Laboratories, Abbott Park, Illinois, USA), a rapid in vitro diagnostic test for quantitative determination of CRP with a measuring range of 5–200 mg/l and an assay time of 3–4 min.

### Statistical analyses

This study was based on post-hoc analyses of data collected for an aetiological study [9], which was originally sized to detect a 10% prevalence difference of *F. necrophorum* between patients and controls with a power of 0.8 and an alpha value of 0.05. Data were analysed using SPSS 25.0 software (IBM, Armonk, NY, USA). For comparison of independent categorical data, we used a two-sided Pearson Chi-squared test or Fisher’s exact test. P-values < 0.05 were considered significant. CRP was described with median value and interquartile range (IQR) due to non-normal distribution, small sample sizes, and a constricted measurement range (5–200 mg/l). Per clinical practice, CRP values < 5 were regarded as 0. For comparison of median CRP values and CRP distribution between independent groups, a Mann-Whitney U test was used for two groups and a Kruskal-Wallis H test for three or more groups. Logistic regression was used to calculate odds ratios for the relationship between CRP value and antibiotic prescribing in patients with a negative RADT. Confidence intervals for sensitivity and specificity were calculated using the binomial (Clopper-Pearson) “exact” method. Predictive values for GAS were reported both as crude values, based on the number of patients with GAS who were tested with CRP, and as adjusted values weighted for the 30% prevalence of GAS in this study [9]. Confidence intervals for positive and negative predictive values were calculated as standard logit confidence intervals according to Mercaldo et al. [40]. Receiver operating characteristic (ROC) curves with area under the curve (AUC) were calculated to evaluate the diagnostic performance of a CRP test to detect GAS in culture, with the optimal cut-off calculated using Youden’s index. The Centor score was calculated as one point each for fever, lymphadenitis, tonsillar coating, and absence of cough, with a maximum of four points [12]. In line with the previous studies in this project [9, 41, 42], only patients with a positive culture for GAS were considered as positive for GAS, regardless of RADT result. Due to the low prevalence of single pathogens, we grouped them for some of the analyses.

## Results

### Use of point-of-care tests

The use of RADTs for GAS and CRP tests is presented in Table 1. RADTs were used in 94% of the patients and equally often at all levels of Centor score. A CRP test was used in 50% of the patients but more commonly in those with a negative RADT (59%) than in those with a positive RADT (38%) (*p* = 0.005). In patients with a positive RADT, a CRP test was used equally often at all levels of Centor score. However, in patients with a negative RADT, a CRP test was used more often in patients with low Centor scores than in patients with high scores. Characteristics of patients tested with CRP are displayed in Table 2.

**Table 1:** Relation between the Centor score, use of point-of-care tests, and antibiotic prescribing in 220 young adults with a sore throat, n (%)

		RADT for GAS			CRP				
Centor score	Number of patients	Patients tested	Positive result		Patients tested	Patients with a positive RADT that were tested	Patients with a negative RADT that were tested	Patients with no RADT that were tested	Antibiotic prescribing
0	16/220 (7)	15/16 (94)	3/15 (20)		9/16 (56)	1/3 (33)	8/12 (67)	0/1	3/16 (19)
1	50/220 (23)	46/50 (92)	5/46 (11)		34/50 (68)	3/5 (60)	29/41 (71)	2/4 (50)	9/50 (18)
2	69/220 (31)	67/69 (97)	18/67 (27)		37/69 (54)	8/18 (44)	28/49 (57)	1/2 (50)	25/50 (36)
3	54/220 (25)	52/54 (96)	24/52 (46)		24/54 (44)	9/24 (38)	15/28 (54)	0/2	30/54 (56)
4	31/220 (14)	27/31 (87)	18/27 (67)		7/31 (23)	5/18 (28)	2/9 (22)	0/4	28/31 (90)
Total	220 (100)	207/220 (94)	68/207 (33)		111/220 (50)	26/68 (38)	82/139 (59)	3/13 (23)	95/220 (43)
*p*	< 0.001^a^	0.3^b^	< 0.001		< 0.001	0.3	0.02	0.4^b^	< 0.001

RADT = Rapid antigen detection test; GAS = group A streptococci; CRP = C-reactive protein (point-of-care test).

*p*-values are for the comparison of Centor score 0–4 and refer to the Cochran-Mantel-Haenszel trend test if not otherwise stated.

^a^ Chi-square test; ^b^ Fisher’s exact test.

**Table 2:** Characteristics of patients tested or not tested with CRP (n = 220)

	CRP	No CRP	*p*
Number of patients (%)	111/220 (50)	109/220 (50)	0.9
Female, n (%)	71/111 (64)	70/109 (64)	1
Age, years, median (IQR)	35 (26–39)	31 (21–38)	0.02^a^
Days with symptoms, median (IQR)	4 (3–7)	3 (2–6)	0.01^a^
Tested with a RADT for GAS, n (%)	108/111 (97)	99/109 (91)	0.04
Positive RADT, n (% of RADTs)	26/108 (24)	42/99 (42)	0.005
Centor score, n (%)			0.002
0	9/111 (8)	7/109 (6)	
1	34/111 (31)	16/109 (15)	
2	37/111 (33)	32/109 (30)	
3	24/111 (22)	30/109 (28)	
4	7/111 (6)	24/109 (22)	

RADT = Rapid antigen detection test; GAS = group A streptococci; CRP = C-reactive protein (point-of-care test).

*p*-values refer to Chi-square test if not otherwise stated.

^a^ Mann-Whitney *U* test.

### Outcomes of point-of-care tests

As previously described, 68/207 (33%) RADTs were positive for GAS and the proportion of positive tests increased with each Centor score [41] (Table 1). Eleven patients with a positive RADT had a negative culture for GAS, and 7 patients with a negative RADT had a positive culture, resulting in a sensitivity of 89% (95% CI 79–95) and a specificity of 92% (95% CI 87–96) [41].

The median CRP value of all tested patients was 15 mg/l (Table 3). CRP was higher in patients with a positive RADT than in patients with a negative RADT (median 41 vs. 9 mg/l; *p* < 0.001). CRP increased with each Centor score both for all tested patients (*p* < 0.001) and for patients with a negative RADT (*p* < 0.01) although not in the 26 patients with a positive RADT (*p* = 0.09).

**Table 3:** Median CRP values at different Centor scores in relation to RADT outcome

	All patients tested with CRP					Patients with a positive RADT					Patients with a negative RADT			
Centor score	No. of patients	Median CRP (mg/l)	IQR	Min–max		No. of patients	Median CRP (mg/l)	IQR	Min–max		No. of patients	Median CRP (mg/l)	IQR	Min–max
0	9	0	0–16	0–26		1	0	0	0		8	0	0–21	0–26
1	34	8	0–24	0–87		3	9	0–9	0–25		29	6	0–23	0–87
2	37	12	0–32	0–200^a^		8	41	24–70	7–89		28	8	0–22	0–200^a^
3	24	32	13–79	0–200^a^		9	46	25–143	10–200^a^		15	25	8–66	0–120
4	7	87	54–194	0–200^a^		5	87	41–167	0–194		2	127	54–200^a^	54–200^a^
Total	111	15	0–36	0–200^a^		26	41	20–88	0–200^a^		82	9	0–26	0–200^a^

CRP = C-reactive protein; CI = confidence interval; IQR = interquartile range.

^a^A CRP value of 200 mg/l was the upper limit of the point-of-care test used in this study.

### Antibiotic prescribing

**Fig. 1:**
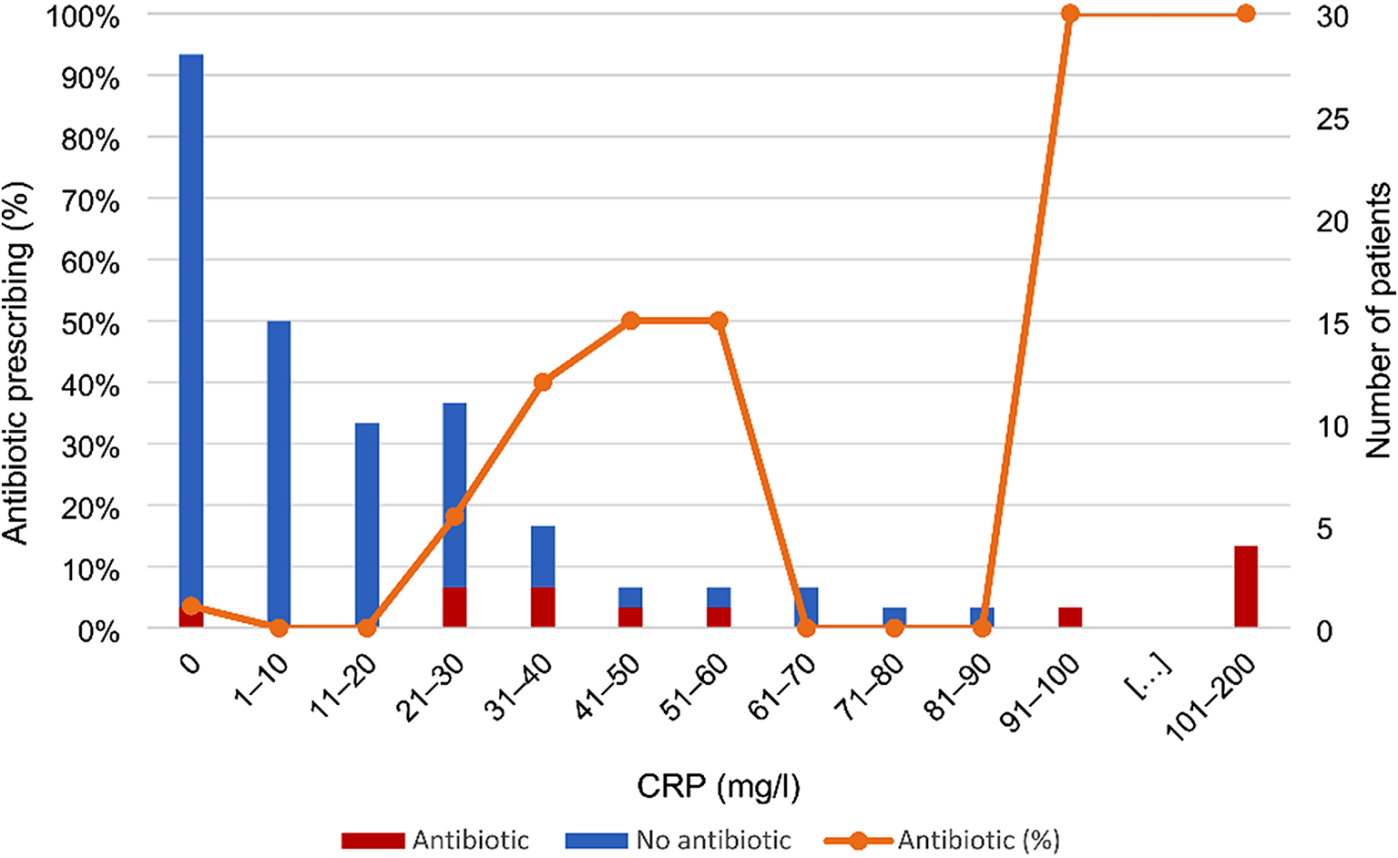
ID=“Par25”>Association between C-reactive protein (CRP) value and antibiotic prescribing (dotted line) in patients with a negative rapid antigen detection test (RADT) for GAS (n = 82). The stacked bars represent the number of tested patients with and without antibiotic prescribing

At the visit, 43% of the patients were prescribed an antibiotic (Table 1). The prescribing differed greatly between patients with a positive RADT (65/68; 96%) and patients with a negative RADT (23/139; 17%) (*p* < 0.001). For patients with a negative RADT, antibiotic prescribing was equally common in patients tested with CRP (12/82; 15%) and patients not tested with CRP (11/57; 19%) (*p* = 0.5). However, for the 82 patients with a negative RADT who were tested with CRP, there was a positive association between CRP value and antibiotic prescribing (OR 1.05; 95% CI 1.02–1.07; *p* < 0.001). Patients with CRP values ≤ 30 mg/l were seldomly prescribed antibiotics (3/64; 5%) compared to patients with CRP values of 31–200 mg/l (9/18; 50%) (*p* < 0.001). Figure 1 depicts the association between CRP value and prescribing in patients with a negative RADT. Overall, 28/31 (90%) patients with a Centor score of 4 were prescribed antibiotics, although only 18/31 (58%) had a positive RADT (Table 1).

### CRP values for different aetiologies

**Fig. 2:**
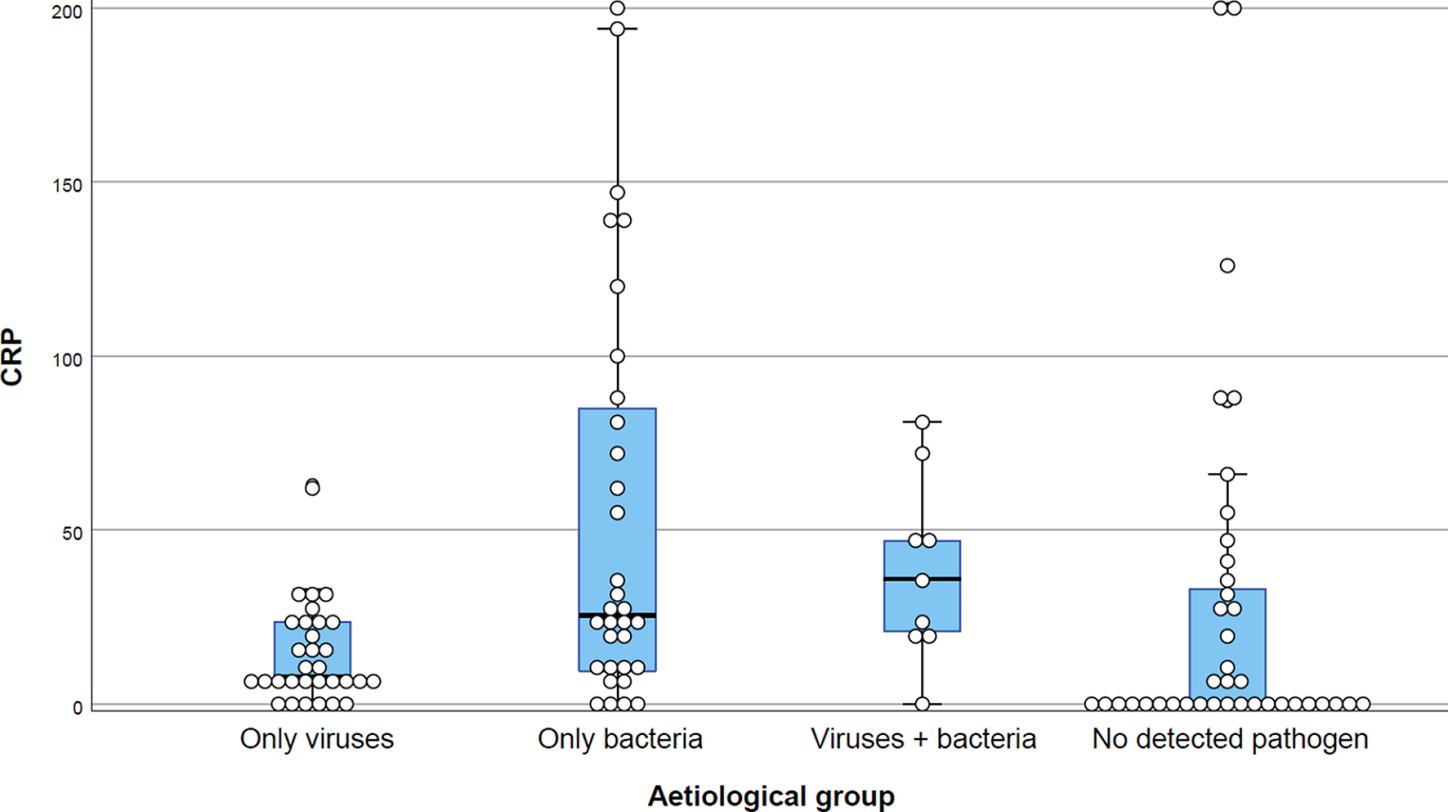
ID=“Par29”>Distribution of C-reactive protein (CRP) values (mg/l) in 111 patients with different aetiological findings. The groups are mutually exclusive and exhaustive. The boxes contain values between the 25th and 75th percentile. The point-of-care CRP test used in this study had a measuring range of 5–200 mg/l and values < 5 were regarded as 0

The CRP distribution and median values for different aetiologies are presented in Table 4; Fig. 2. Patients with GAS in culture had a median CRP value of 46 mg/l, compared to 8 mg/ml in patients without GAS (*p* < 0.001). As GAS was the most prevalent pathogen in this study it highly influenced CRP values in the groups “any bacteria”, “only bacteria”, and “any streptococci”. Patients with only viruses had a lower median CRP than patients with only GAS (8 vs. 35 mg/l; *p* = 0.001) and patients with only bacteria (8 vs. 26 mg/l; *p* < 0.002). CRP differed between the groups “only viruses”, “only bacteria”, “viruses + bacteria”, and “no detected pathogen” (*p* = 0.001) (Fig. 2). The lowest median CRP was seen in patients with no detected pathogen.

**Table 4:** Median CRP values in relation to aetiological findings (n = 111)

Aetiology	Patients in total, n (%)	Patients with CRP	Days with symptoms, median (IQR)	CRP (mg/l), median (IQR)
Any bacteria	103/220 (47)	41/103 (40)	4 (3–5)	26 (11–77)
Only bacteria	85/220 (39)	32/85 (38)	3 (3–5)	26 (9–87)
Any GAS	66/220 (30)	25/66 (38)	4 (3–4.8)	46 (17–95)
Only GAS	46/220 (21)	17/46 (37)	3 (3–4.8)	35 (17–139)
No GAS in culture	154/220 (70)	86/154 (56)	5 (3–7)	8 (0–26)
Any streptococci (A/C/G)	81/220 (37)	31/81 (38)	3 (3–4.3)	31 (9–89)
Any non-GAS bacteria	37/220 (17)	16/37 (43)	3.5 (3–5.3)	20 (9–33)
Any group C/G-streptococci	15/220 (7)	6/15 (40)	3 (3–6)	13 (5–46)
Any *F. necrophorum*	33/220 (15)	14/33 (42)	4 (3–5)	23 (11–58)
Only viruses	52/220 (24)	31/52 (60)	5 (3–7)	8 (5–24)
No detected pathogen	65/220 (30)	39/65 (60)	6 (3–8.5)	0 (0–36)

CI = confidence interval; CRP = C-reactive protein (point-of-care test); GAS = group A streptococci; IQR = interquartile range.

Aetiological data was based on throat culture, PCR and serology, but not on outcomes of rapid antigen detection tests. “Only” and “any” refers to findings with or without a concomitant presence of one or more of the other sought-after pathogens (see Methods).

### CRP and aetiological prediction

The properties of CRP as a diagnostic test for GAS, using culture as the reference, are presented in Table 5. In ROC analysis the AUC was 0.75 (95% CI 0.64–0.87) and the optimal cut-off was determined to be CRP 22 (sensitivity 0.76 and specificity 0.69). However, due to the high prevalence of other pathogens, PPVs for GAS reached a maximum of 0.60 (95% CI 0.31–0.83) at the investigated CRP levels and only increased marginally when adjusted for the 30% prevalence of GAS found in this study.

**Table 5:** Sensitivity, specificity, PPV and NPV of CRP as a test for detecting GAS (n = 111)

CRP (mg/l)	No of patients	GAS	No GAS	Sensitivity (95% CI)	Specificity (95% CI)	PPV (95% CI)	NPV (95% CI)	Adjusted PPV*	Adjusted NPV*
≥ 0	111	25	86	1 (0.86–1)	0 (0–0.04)	0.23 (0.23–0.23)	N/A	0.30 (0.30–0.30)	N/A
≥ 10	61	20	41	0.80 (0.59–0.93)	0.52 (0.41–0.63)	0.33 (0.27–0.40)	0.90 (0.80–0.95)	0.42 (0.35–0.49)	0.86 (0.73–0.93)
≥ 20	50	19	31	0.76 (0.55–0.91)	0.64 (0.53–0.74)	0.38 (0.30–0.47)	0.90 (0.82–0.95)	0.48 (0.38–0.56)	0.86 (0.75–0.93)
≥ 30	34	15	19	0.60 (0.39–0.79)	0.78 (0.68–0.86)	0.44 (0.32–0.57)	0.87 (0.80–0.92)	0.54 (0.41–0.67)	0.82 (0.74–0.88)
≥ 40	26	13	13	0.52 (0.31–0.72)	0.85 (0.76–0.92)	0.50 (0.35–0.65)	0.86 (0.80–0.90)	0.60 (0.44–0.73)	0.80 (0.73–0.86)
≥ 50	22	11	11	0.44 (0.24–0.65)	0.87 (0.78–0.93)	0.50 (0.33–0.67)	0.84 (0.79–0.88)	0.60 (0.42–0.75)	0.78 (0.72–0.84)
…									
≥ 100	10	6	4	0.24 (0.09–0.45)	0.95 (0.89–0.99)	0.60 (0.31–0.83)	0.81 (0.78–0.84)	0.69 (0.40–0.88)	0.75 (0.70–0.79)

CI = Confidence interval; CRP = C-reactive protein; GAS = group A streptococci; NPV = negative predictive value; PPV = positive predictive value.

* Assuming a 30% prevalence of GAS (in accordance with the aetiological findings of this study [9]) with the same distribution of CRP values as in the tested patients.

Table 6 presents crude PPVs for “any GAS”, “any bacteria”, and “only viruses”. CRP was generally more predictive of “any bacteria” than of “any GAS” regardless of CRP level. PPVs for “any bacteria” were only moderate at all investigated CRP levels, even in patients with CRP ≥ 50 mg/l. However, these PPVs were not adjusted for the lower rate of CRP testing in patients with bacteria compared to patients with “only viruses” or “no detected pathogen” (Table 4) but based on the factual use of CRP in this study. PPVs for “only viruses” diminished rapidly with rising CRP levels, and was only 0.12 when CRP ≥ 30 mg/l.

**Table 6:** PPV for “any GAS”, “any bacteria”, and “only viruses” at different levels of CRP (n = 111)

		Any GAS				Any bacteria				Only viruses		
CRP (mg/l)	No. of patients	Pos	Neg	PPV (95% CI)		Pos	Neg	PPV (95% CI)		Pos	Neg	PPV (95% CI)
≥ 0	111	25	86	0.23 (0.23–0.23)		41	70	0.37 (0.37–0.37)		31	80	0.28 (0.28–0.28)
≥ 10	61	20	41	0.33 (0.27–0.40)		32	29	0.52 (0.44–0.60)		21	40	0.34 (0.27–0.42)
≥ 20	50	19	31	0.38 (0.30–0.47)		27	23	0.54 (0.44–0.64)		10	40	0.20 (0.13–0.30)
≥ 30	34	15	19	0.44 (0.32–0.57)		19	15	0.56 (0.42–0.69)		4	30	0.12 (0.05–0.26)
≥ 40	26	13	13	0.50 (0.35–0.65)		16	10	0.62 (0.45–0.76)		1	25	0.04 (0.006–0.22)
≥ 50	22	11	11	0.50 (0.33–0.67)		14	8	0.64 (0.45–0.79)		1	21	0.05 (0.007–0.25)
…												
≥ 100	10	6	4	0.60 (0.31–0.83)		7	3	0.70 (0.39–0.90)		-	10	-

CI = Confidence interval; CRP = C-reactive protein; GAS = group A streptococci; NPV = negative predictive value; PPV = positive predictive value.

### CRP in patients with Centor score 3–4 and a negative RADT for GAS

Of the 82 patients with a negative RADT and a CRP test, 17 had a Centor score of 3–4. The aetiology in these patients were “only viruses” (n = 6), “only bacteria” (n = 6), “viruses + bacteria” (n = 1), and “no detected pathogen” (n = 4). The identified bacteria were “only *F. necrophorum*” (n = 3), “only GAS” (n = 1), “only group G streptococci” (n = 1), and “group G streptococci + *F. necrophorum*” (n = 1).

**Fig. 3:**
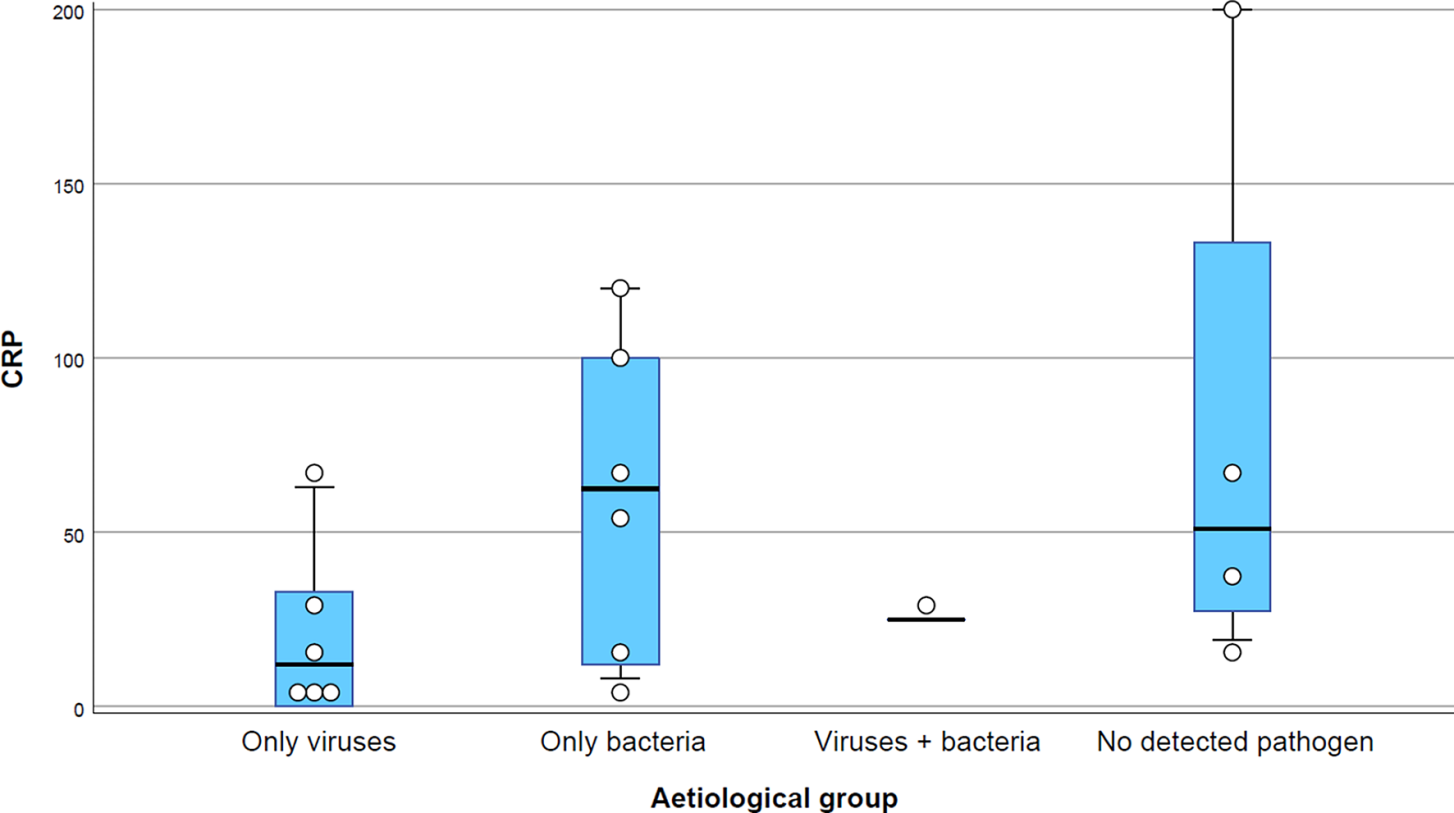
ID=“Par37”>Distribution of C-reactive protein (CRP) values (mg/l) for different aetiologies in 17 patients with a Centor score of 3–4 and a negative rapid antigen detection test (RADT) for group A streptococci (GAS). The groups are mutually exclusive and exhaustive. The boxes contain values between the 25th and 75th percentile. The point-of-care CRP test used in this study had a measuring range of 5–200 mg/l and values < 5 were regarded as 0

Patients with “only viruses” had a median CRP of 12 mg/l (IQR 0–12), patients with “only bacteria” had a median CRP of 63 mg/l (IQR 11–105), the one patient with “viruses + bacteria” had a CRP value of 25 mg/l, and patients with no detected pathogen had a median CRP of 51 mg/l (IQR 23–167) (*p* = 0.2) (Fig. 3). The outlier CRP value of ≥ 200 mg/l belonged to a patient with no detected pathogen and a clinical diagnosis of incipient peritonsillitis. The difference in CRP between patients with “only viruses” and “only bacteria” was not statistically significant (*p* = 0.09). Neither was it between the seven patients with “any bacteria” (median CRP 54 mg/l; IQR 12–100) and the 10 patients with “no bacteria” (median CRP 26 mg/l; IQR 5–64) (*p* = 0.36). Due to the similar CRP distribution in patients with “any bacteria” and patients with no detected pathogen, the PPV for “any bacteria” if CRP ≥ 20 mg/l was only 0.50 (95% CI 0.32–0.69). Conversely, the PPV for “only viruses” if CRP ≤ 20 mg/l was a moderate 0.57 (0.30–0.80).

## Discussion

### Main findings

In this observational study on point-of-care tests in patients with pharyngotonsillitis in PHC, we saw a high use of both RADTs and CRP tests, which is a huge deviation from the national guideline. The majority of CRP tests were used in patients with a negative RADT. Almost all patients with a positive RADT were prescribed antibiotics, but many patients with a negative RADT were also prescribed antibiotics if the CRP value exceeded 30 mg/l. CRP levels were significantly higher in patients with GAS compared to patients without GAS, but the PPVs for GAS at the examined CRP levels were too low to be clinically useful.

### Strengths and weaknesses

As the self-referred and nurse-triaged population of this study reflects everyday practice in Swedish urban PHC centres, where patients present a heterogenous spectrum of symptoms and disease severity, the results should be generalisable to PHC centres both within and outside Region Kronoberg. Another strength is that we had access to broad clinical data, including the Centor score, as well as a detailed analysis of both bacterial and viral aetiology, which contrasts registry-based studies. Registries, however, can provide much larger sample sizes than observational studies and be more extensive in terms of the number of study sites. The data in this study were collected many years ago and behavioural patterns may have changed since then, but this also means that the study was not affected by the COVID-19 pandemic. The greatest limitation of this study is the post-hoc analysis, as the analysis of point-of-care tests was not planned in advance. This raises some questions that cannot be fully answered. The first concern is that the awareness of being observed (i.e. the Hawthorne effect) may have prompted some doctors to examine the patient more thoroughly, including more frequent use of POC tests; however, one might also expect the Hawthorne effect to work in the opposite direction, making doctors more likely to follow guidelines. Moreover, we do not know whether the included PHC centres were high-prescribing practices, and we do not know the age and gender of the participating doctors, characteristics that can influence the likelihood of POC testing [43]. The second concern is that we do not know which POC tests were ordered at the same time and which were ordered sequentially, which makes it difficult to interpret doctors’ behaviours. The third concern is that the CRP tests were not ordered randomly as they were more often ordered for patients with a negative RADT for GAS and therefore in patients with slightly lower Centor scores. As a result, fewer patients with GAS than without GAS were tested with CRP.

### Interpretation

#### Behaviour

Surprisingly, RADTs were used in almost all patients, although only 39% of patients had a Centor score of 3–4, which is the selection criterion for a RADT in the Swedish guideline [15]. Perhaps even more surprisingly, a CRP test, which the guideline explicitly advises doctors not to use [15], was used in half the patients. CRP testing was more common in patients with a negative RADT than in patients with a positive RADT, suggesting that the two tests were often used sequentially. However, since 38% of patients with a positive RADT were also tested with CRP, it is likely that many of these tests were ordered at the same time in advance. Paradoxically, in patients with a negative RADT, CRP testing was used in patients with low Centor scores – generally suggestive of non-bacterial aetiology – more often than in patients with high scores.

The widespread use of POC tests reflects a general lack of adherence to guidelines, as has been shown elsewhere [24, 32, 37, 38]. Some factors that have been used to explain poor adherence are outdated knowledge, distrust of test results, diagnostic uncertainty, and difficulties refusing patients’ requests for a prescription without the support of a negative test result [38, 43]. The proportion of patients tested with CRP in this study is consistent with recent results from studies in Denmark [24, 37] and is also in line with the current use of CRP tests in Region Kronoberg, where 45% of patients diagnosed with pharyngotonsillitis in PHC during the last year were tested (data extracted 2022-10-07 from PHC records with Medrave 4, Medrave Software AB, Stockholm, Sweden). However, one difference between Denmark and Sweden is that many general practitioners in Denmark, unlike in Sweden, are reimbursed by the National Health Service for these tests and therefore have a financial incentive to perform POC tests [43].

A positive RADT was virtually synonymous with antibiotic prescribing, which is consistent with the guideline [15] and logical as testing would otherwise be redundant. On the other hand, RADTs were used excessively at all levels of Centor scores and 38% of patients with a positive RADT had a score of 0–2. These patients not only receive no proven benefit from antibiotics but also are at risk of medicalization, which alters their expectations for testing future episodes of pharyngotonsillitis [14]. CRP did not affect antibiotic prescribing in the light of a positive RADT. In contrast, in patients with a negative RADT, there was a positive association between CRP level and antibiotic prescribing, and patients with CRP < 30 mg/l were unlikely to be prescribed antibiotics. However, compared with patients who were not tested, CRP testing did not reduce antibiotic prescribing at the group level. That is, CRP testing sometimes led to prescribing that would not otherwise have been done. This contrasts with the argument that CRP tests can help reduce antibiotic prescribing for respiratory tract infections [26]. The positive association between CRP level and antibiotic prescribing is in line with previous results from Denmark, where 75% of patients with an upper respiratory tract infection were prescribed antibiotics if their CRP was > 50 mg/l [24]. According to a Cochrane review, RADTs can reduce prescribing in patients with pharyngotonsillitis by as much as 25% (18–31%) [18]. This could be true in an ideal situation where doctors follow the guidelines. However, in this study, antibiotics were prescribed to 19% of patients with a negative RADT and no CRP test, and as mentioned above, many patients with low Centor scores were tested with an RADT and then prescribed antibiotics if the test was positive. A Centor score of 4 also led to antibiotic prescribing in most cases regardless of test results. If only patients with a Centor score of 3–4 and a positive RADT had been treated, the prescribing rate of this study would have been 20% rather than to the observed rate of 43%.

#### Aetiology

The median CRP value differed between aetiologies. The highest value was observed in patients with GAS and this was followed by patients with “any bacteria”. The lowest value was observed in patients with no detected pathogen. A large proportion of the patients in this study had no detectable pathogen despite a clinical diagnosis of infectious pharyngotonsillitis, suggesting a problem with sampling techniques or detection methods. This is further illustrated by CRP values > 100 mg/l in some of these patients, which could indicate a bacterial aetiology. The difference in CRP values between bacteria and viruses was expected [27–31, 35], although some studies have failed to show such a difference [32, 33]. However, a statistically significant difference is only of interest if it is also clinically meaningful. Unfortunately, the PPVs for GAS and for bacteria as a group were only moderate at best, making it very hard for the clinician to assess an arbitrary CRP value. In patients with a Centor score of 3–4 and a negative RADT, the suspicion of bacterial aetiology may be heightened; however, in this study, the great overlap of CRP values between bacteria and no detected pathogen resulted in low to moderate predictive values even in this group. Interestingly, the CRP value increased with each Centor step, making CRP an expensive and cumbersome way to measure the Centor score. CRP has been proposed as a test for GAS in low-resource settings where RADTs are not available [28, 44, 45]; in this study, we found the sensitivity to be 0.8 at CRP ≥ 10 mg/l, which would result in treatment of about half of the patients if this level was chosen as the cut-off. This proportion is consistent with a previous report from Thailand [28].

## Conclusions

The use of point-of-care tests in this study was high and shows poor adherence to the national guideline. RADTs were used indiscriminately in almost all patients, and a CRP test was used in a majority of patients with a negative RADT. A positive RADT resulted in antibiotic prescribing in most cases, while the CRP value seemed to govern antibiotic prescribing in patients with a negative RADT, suggesting a perceived need for antibiotics with increasing levels of CRP. We found an association between aetiology and median CRP value, with patients with GAS having the highest value and patients with viruses or no detected pathogen having the lowest values. However, the positive predictive values of CRP for GAS and for any bacterial finding, respectively, were too low to be clinically useful.

## Data Availability

The datasets used and/or analysed during the current study are available from the corresponding author on reasonable request.

## List of abbreviations

CI: Confidence interval
CRP: C-reactive protein
GAS: Group A streptococci
IQR: Interquartile range
PHC: Primary health care
POC: Point of care
PPV: Positive predictive value
RADT: Rapid antigen detection test
